# On the Pathology and Treatment of Epidemic Cholera

**Published:** 1849-12

**Authors:** 


Art. VI.—*On the Pathology and Treatment of Epidemic Cholera.* By Samuel Annan, M. D., Professor of the Theory and Practice of Medicine, in Transylvania University.

An article, bearing the above caption, appeared in the last number of the Transylvania Medical Journal. The position of the writer—a teacher of practical medicine in the oldest medical institution west of the mountains—and the character of the essay—singular for its claims to originality in the use of a very old and common practice—must be our excuse, in presenting thus, parts of it to the notice of our professional brethren. Nor shall we confine ourselves entirely to this one article of Prof. A.'s, for shortly after his transfer from the chair of Obstetrics, to that of Theory and Practice, and during the prevalence of cholera in Lexington, so anxious was he to give advice, over his new professorial titles, to the profession (or public ?) that, not able to await the appearance of a medical journal, he sent to one of the political semi-weekly papers of that place, a half column of matter relative to the management of the disease then raging, and in about two weeks afterwards, through the same medium, repeated the dose. To each of these pieces, we feel compelled to allude. That the Professor of Theory and Practice in the Transylvania medical school, should have leisure, during the prevalence of a wide spread and malignant epidemic, and at a time when all the physicians of the place were occupied night and day, to concoct newspaper paragraphs—needs no comment. But to the pieces in question:—

After a few desultory remarks, concerning the causes of cholera, and its contagious or non-contagious character, etc., we meet with the following sentence, containing a doctrine so opposed to our own observation, that we must dwell upon it for an instant. "In contradistinction from this, [typhoid premonitaries] with a few exceptions, those attacked by cholera are in comparative health prior to the commencement of the first stage of the disease." Which first stage is, according to Prof. A., "feculent diarrhea."

This is certainly an ambiguous sentence, and may be construed either in favor of, or adverse to the existence of premonitory choleraic symptoms, as may best suit Prof. A.'s convenience. What are we to understand by "comparative health"? Is it meant that a person is well enough to be about, whenever not sufficiently sick to lie down, or does it mean that, alike from the absence of all the signs and symptoms of disease, as from any information to be derived from the patient, we are unable to foresee or forestall a threatened attack? This last seems to us the most natural interpretation, and particularly so, as in the sentence preceding the one under consideration, he makes mention of the "malaise, languor, lassitude and a feeling of weakness and weariness," which usually usher in a case of typhoid fever.

Well, what, according to authors and according to facts, is the true state of the case?

Mr. Gendrin, in his clinical lecture upon this disease, published in the Gazette des Hôpitaux, March 31, 1849, says that they are always present (Le choléra est tonjours précédé de prodromes,) and moreover, that they sometimes commence as long as eight days beforehand. Mr. Tanchou, in the Society of Practical Medicine, during its meeting, held at Paris, on the 5th of July, said he had never seen a case "absolument exempt de prodromes." M. Chomel, the Professor of Clinical Medicine in the Paris school, than whom no one is more extensively known to the profession everywhere, and whose opinion most justly bears the great weight it deserves, admits that the disease is sometimes foudroyant, or most sudden in its attack, but this is the exception and not the rule, for he says:* "Sans doute, le plus souvent, les choses se passent ainsi: il y a des prodromes qui précedent de quelques jours l'invasion des symptoms cholériques." "Les premiers phenomines d'invasion sont d'ailleurs assez variables, elles se manifeste chez quelques individus, par la sensation d'un poids qui les accable. D'autres, etc., etc., Dans l'epidémie de 1832, la sensation la plus commune était celle d'une barre qui traversait l'épigastre." Mr. Rostan† makes five periods:—1. Prodromie. 2. Invasion. 3. Algide. 4. Reaction. 5. Return to health.— Dr. Bell‡ says: "On an early attention to the premonitory symptoms, or to this first or forming stage of the disease, will greatly depend the favorable issue of the case." Prof. Lawson,§ of Cincinnati, in his excellent résumé of all that has been written upon Asiatic cholera, says:— "The history of cholera in every country shows that it is almost invariably preceded by certain premonitory symptoms."

---

* Gazette des Hôpitaux, April 3.
† Gazette des Hôpitaux, May 24.
‡ Bell & Stokes' Practice, vol. 1, p. 381.
§ Western Lancet, vol. viii, no. 6, p. 344.

So far as we have been able to push our investigations, we have yet to see the first writer, Prof. A. excepted, who does not, either directly or indirectly, assert the existence of the premonitory stage—some of them asserting with Mr. Gendrin that it is invariably present, but all admitting its existence in the large majority of cases. In a word, what is the meaning of the lassitude, the gastric embarrassment, the gargouillement and abdominal tension, the feeling of weight and oppression over the epigastrium, the disordered bowels, the depression of spirits, and many other symptoms of a deranged condition of the system, if they are not premonitories of an attack? True, these may exist in many cases without being followed by serious consequences, but so do clouds often lower upon the horizon, and scatter without a storm.

"The milk-white tongue is very peculiar, and in proportion to the thickness of this covering, is the predisposition to an attack. During the prevalence of the epidemic in this city, the tongue of most persons exhibited more or less of this appearance; and this was especially the case with those whose healthful feelings were somewhat disturbed."—This, we regret to say, is entirely opposed to our own experience and observation. Nor is this all—since the publication of Professor A.'s article we have made inquiries of several of the practitioners in Lexington, and especially those whose field of action was the most extensive, and find our observation fully sustained by their's. So far as the appearance of the tongue went, we hazard nothing in saying that it was indicative of naught, either before, during, or after the termination of an attack of cholera, and this was true alike in mild and severe cases, as it appeared during the past summer. And furthermore, if there be any other author who makes mention of this milk-white coating of the tongue, as an important characteristic of cholera, we do not now remember who he is. Dr. Parkes, page 97, speaks of a "cool pale tongue," in enumerating the premonitory symptoms of this disease, but we cannot, even by the most forced construction, confound this with a "milk-white" coating.

"The only post mortem appearance which I regard as of any importance, is that presented by the blood."

In this article the Professor does not give the details of a single autopsy, nor in fact does he lay claim to the credit of having made one himself, or having ever been present at one, and seen the inside of a defunct cholera patient. That his opinions upon this point, at least, of his subject, are purely speculative, those who are aware of his ultra contagious views—which, by-the-bye, he most studiously, for reasons well known to himself and to the medical profession in Lexington, avoids expressing in the article before us—do not doubt. That the abnormal state of the blood constitutes a great element in the pathological lesions of cholera, we will not deny—though were we disposed so to do, we might quote, in support of our opinion, the following sentence from M. Chomel :* " Quant au sang, il ne pré[illegible]ent pas d' alteration assez nette ni assaz constante pour qu'au simple aspect [illegible] puisse l' attribuer au cholera."

But whether the change in this fluid is the cause or the effect of the disease, may admit of debate. For our part, we are not sufficiently biassed by humoralism to believe that healthy solids elaborate abnormal fluids. Though Prof. A. does assert that, " few members of the medical profession will deny, that it [epidemic cholera] operates upon the human organism, by being introduced into the blood, and in this way circulating through all the organs and tissues, produces its peculiar effects." In fact, to affirm gravely that " the only post mortem appearance" " of any importance, is that presented by the blood,"—one must be grossly ignorant of the history of the disease as detailed by the first authorities in the world, and as verified by necropsic examinations, or else set out with the desire of basing a reputation for originality and profundity upon that most miserable of all foundations, unsustainable assertion. Is the shrivelled, shrunk condition of the body to go for nothing? Are the contents of the alimentary canal, the emptiness of the bladder, and its contracted walls undeserving of attention? Is the dryness of the serous surfaces to be passed by without so much as an allusion? Do not the small and hardened spleen, the softened, congested and friable heart, the congestion of the nervous system, and the condition of the lungs, liver, and kidneys deserve mentioning? If Professor A. had deigned to consult the works of those, at least his equals in professional acquirements, or, still better, had made investigations in the dead-room, he would never have given utterance to the opinion that " the only *post mortem* appearance" " of any importance, is that presented by the blood."

" The mucous coat is pale, except where there is great capillary congestion and ecchymosis, as in the more depending parts; and it requires a microscope to discover hypertrophy of the follicles." M. Grisalle, in his masterly work, says :† " La memb[illegible]a[illegible]e mu[illegible]ueuse a gnéralement sa

* Gazette des Hôpitaux, April 14, 1849.
† Pathologie Interne, vol. i, page 724.

couleur et sa consistence normales; mais elle présente dans toute l'etendue des voies disgestives, depuis l'œsophage jusqu'au rectum, et spécialement à la fin de l'ileon, dans le cœcum et le colon, une éruption de petits corps durs et opaques, résistant à la pression, du volume d'une tête d'épingle ou d'un grain de chénevis ou de mil, impénétrables aux injections." M. Serres, at a meeting of the Institute of France, on the 9th of April, 1849, remarked :* " En 1832, j'ai fait ouvri'r de 60 à 80 cadavres de choleriques dès le début de l'épidèmie, et j'ai [illegible] frappé de la constance de cette éruption psorentérique, que j'ai trouvé sans exception chez tous les malades." M. Chomel† regards this as one of the principal lesions in cholera. M. Gendrim‡ says that these " microscopic bodies, as large as a grain of millet, are sometimes so thickly developed on the intestinal mucus that they seem to have been sown broadcast (été jétés à poignée). Dr. Parkes,§ of London, who, in his monograph upon cholera, gives minutely the post mortem appearances drawn from forty-six dissections, reports: " The agminated and solitary glands were enlarged in a majority of cases." " In 22 cases the agminated glands were considerably, and, in some cases, greatly enlarged." "The solitary glands were much enlarged in 16 cases." " When the Peyerian glands were small, the solitary glands were generally well developed; and the reverse"—in the large intestines, " the glands were visible in every case." Enough has certainly been said on this point, for though authorities without number might be quoted, all going to prove the idleness of Prof. A.'s assertion, if the weight of testimony here offered is not against him, and most of the gentlemen named above, stand on the uppermost round of the ladder of professional merit, it is useless to discuss it further. For our part, we regret to learn that the Professor's eyes are so dimmed (by age?) that he needs a microscope to see objects the size of a pin's head, but, at the same time, his candor in admitting it is highly commendable. So much for the pathology of epidemic cholera—let us now turn to the treatment proposed by Prof. A.

" No better evidence is required of the facility with which the feculent diarrhea, by some called precursory, can be arrested, than the unquestionable fact, that such a vast variety of means have been found sufficient for this purpose." " In the first stage ['feculent diarrhea'] I have given the following powders: R. Calomel, ʒss., plumb. acet. ʒss., pulv.

* Gazette des Hôpitaux, April 10, 1849.

opii. grs. x., pulv. capsici vel camphor ℈j. M. Divide in pulv. x. signa. One powder every half hour, mixed in syrup, until three or four are taken." Does not this seem an uncalled for waste of medicine? Where is the propriety of giving from nine to twelve grains of calomel, as much of the sugar of lead, three or four grains of opium, and double that quantity of red pepper, to master a diarrhea which the Professor informs us, has "not unfrequently" been checked, and the patient cured, "by simply irritating a large portion of the skin"? But this is not the only instance on record, in which, by his own admission, Dr A. directs more medicine to be given than is necessary. In his advertisement of the 14th of July, he directs us to "give calomel in small pills by the mouth, until from twenty to sixty grains are retained in the stomach.—Thirty or forty grains of this medicine will do all that this medicine can do." If this is his candid, deliberate opinion, what excuse is there for giving, and advising others to give, from twenty to thirty grains of calomel more than, according to his own admission, can possibly be useful? Why unnecessarily encumber the stomach of your patient, and particularly at a time when it is in a very high degree irritable? After recommending injections of the acetate of lead and laudanum, to check the discharges per ano, and finding them fail, when given in the usual way, he directs as follows: "Here a gum-elastic tube should be passed into the bowels, ten or more inches, the patient lying on his left side, and an injection composed of eighty or one hundred grains of acetate of lead, with two tea-spoonfuls of laudanum, in half a pint of rain water, should be injected into the descending colon, and pressure should be made upon the anus to prevent its escape." The propriety of still further irritating an already highly irritated intestine, by introducing into it, "ten or more inches," a gum-elastic tube, we think at least questionable, and we are so old-fashioned as to believe that this tube may act as a suppository, and excite action in a still greater degree than previously existed. If the injection, after being thrown high up into the colon, runs immediately back to the orifice of the rectum, what is gained by throwing it to this height—for, according to Dr. A.'s views, it must be absorbed before it can exert its astringing and sedative effects? If, on the contrary, it does not run back to the anal opening, why, in the name of common sense, set a nurse to pressing, "fundamentally," (as one of them once expressed himself,) a bundle of wet rags to the patient? If it does not remain where it is thrown, but descends to the sphincter, it is more troublesome and disagreeable to both nurse and patient than an injection administered in the usual way, and possesses over it no advantages. But if it is an improvement upon the common mode, and the enema remains high up in the colon, then is all pressure upon the anus unnecessary, and being unnecessary, a most inexcusable annoyance. But does the tube pass directly up the bowel, as he asserts? It can be no easy matter to pass a flexible, elastic tube "ten or more inches" up the gut, and it is much more probable that it bends and doubles upon itself, than that it passes directly up to the extent described by Prof. A. We have seen several of these tubes, after they had been used, and invariably found them broken and bent as we have described. Upon one occasion, on going in to visit a patient in the last stages of the disease, we found the nurses administering, or rather trying to administer, an injection—after this most approved style—but the fluid escaped from the orifice of the anus in direct ratio to the pressure applied to the piston of the syringe. The tube was, by our direction, withdrawn, presenting more the appearance of a chain saw than a catheter; and an enema, in the usual way, thrown directly into the rectum. In his advertisement of the 1st of August, in addition to the above, Prof. A. directs the injection "to be forced hard, with a syringe through the tube." This, like everything else emanating from the Professor, is of such doubtful utility, to use no harsher term, that we can neither try it ourselves, nor recommend it to others.

In his publication of the 14th July, Prof. A. not only gave his own views with regard to the treatment of cholera, but made a few passing remarks, for the benefit of those members of the profession, who, finding injections of lead and laudanum fail, in many cases, in checking the profuse discharges from the intestines, combined with them the tannic acid. "Every one," he says, "with the smallest knowlege of chemistry, is aware that they [acetate of lead and tannin] are incompatible substances. An insoluble tannate of lead is formed, which is useless, and the acetic acid is highly injurious. No better plan could be devised for keeping up the diarrhea. Warm water, too, is preferable to thin starch, as a vehicle for the lead and laudanum. The object to be attained is their absorption, and the starch is in the way, and causes the absorption to go on more slowly." So the learned Professor hopes, by the publication of views based upon aborted hypothesis, to change the practice of those who have experience for their guide. We are forced to conclude that he has never tried the effect of this combination of chemical incompatibles—but stiil he issues against them his professorial edict, based, not upon his own experience, or that of others, but upon his "smallest knowledge of chemistry." In opposition to his theory, we must beg to record our experience. We have used this compound of lead and tannin, and we used it with as full a knowledge as Prof. A. himself of the chemical incompatibility, but in the school in which we were taught, we learned that medicines might be chemically incompatible, and yet used with the most decided advantage in practice. We used this combination both before and after the proclamation of the 14th July, nor have we any reason to regret it; for we, in almost every case, succeeded in checking the diarrhea, and that, too, after the injections of lead and laudanum had failed. Nor are we alone in this, for it was tried by other physicians of Lexington and the surrounding country—all of whom speak favorably of its effects, and inert as Prof. A. may deem the "insoluble" compound, resulting from the union of sugar of lead and tannic acid—the tannate of lead—Messrs. Trousseau & Pidoux in the first volume of their excellent work on Therapeutics, page 155, place it among the astringents, and speak of its beneficial effects as such. Is Prof. A. aware that the objection, urged by him, against the combination of lead and tannin, exists to a certain extent to his favorite injection of lead and laudanum? Does he know that an insoluble meconate of lead is formed, which is, according to himself, utterly worthless? If he does not know that this chemical decomposition takes place, he is unfitted by ignorance, to teach practical medicine, even to beginners. But if, on the contrary, he is aware of it, then is he a dishonest writer, who prepares his articles as baits for the public, and not with the expectation that they shall pass through the ordeal of medical criticism.

While on the subject of incompatibles we would suggest the fact that Dr. Chapman, in his work on Therapeutics, vol. ii, p. 359, in a note, says: "Certain medicines, too, seem to be incompatible with lead, not from any *chemical* changes, but on account of the *counter* agency which they exercise in their medicinal action. Of this description is mercury, as is illustrated in the effects of that article in saturnine colic," etc.—Now this may, or may not be true—we can hardly suppose that it is, for certainly Prof. A. would not, after all he has said on the subject of chemical incompatibility, use, still worse, medical incompatibles—but then, on the other hand, Dr. Chapman is, to say the least of it, quite as extensively and favorably known, and of as great weight in the profession as is the Professor of Transylvania.

In the same piece, Dr. A. objects to the administration of tannin by the mouth, on account of its producing emesis. During the late epidemic we gave in the form of pill, and saw given by others, thousands of grains of this medicine, and can unhesitatingly assert that it fulfilled, to a higher degree than any other one remedy, our most ardent expectations. Nor was it more offensive to the stomach than the sulphate of quinine, for though some are vomited by quinine as by an emetic, this is the exception and not the rule—the same is true of tannin.

How it happens that astringent and sedative injections have to be absorbed into the system before they commence exerting their peculiar effects upon the parts to which they are applied, we must leave for older and wiser heads than ours to explain, it certainly is opposed to all of our preconceived ideas—possibly Dr. A. can enlighten us. "By the use of large quantities of acetate of lead and laudanum, thrown high into the bowels, through a tube, I have never failed to subdue the serous exhalation." (?) "It has occurred to me that the only safe plan of restoring to the circulation, what has been lost by the serous hemorrhage from the bowels, would be to inject high into the colon, a large quantity of warm water, containing in solution bi-carbonate of soda and chlorate of potash. * * * * * I have thought, that, possibly, by injecting into the colon, two or three quarts of warm water, holding in solution one ounce of the bi-carbonate of soda, and half an ounce of chloride of potash, and retaining this injection by firm pressure on the anus, that absorption would go on with sufficient rapidity to interrupt the sinking process, excite the heart to augmented effort, restore the functional action of the more important organs, and prevent death. That the absorbing veins of the colon are ready to take up whatever is presented to them, of suitable properties, is clear to my mind from what has come under my own observation. After injecting the solution of acetate of lead mixed with laudanum, by means of the tube, my patient has tasted them in from one to two minutes."

The above extracts constitute perhaps the most remarkable parts of the whole paper, remarkable as it is in its totality. In ordinary times a *pint* of warm water injected into the bowels, is, of itself, sufficient to produce an action, yet here we have the grave and learned Professor of a most important branch of medical education, teaching us to inject TWO OR THREE QUARTS of a warm saline fluid, and that, too, in a disease of which purging is one of the most dangerous and troublesome symptoms; but not content with injecting this enormous quantity, he seriously proposes to cause its retention by means of pressure. Wha faith he must have in the power which this favorite means can exert—a means so old* and so useless as to be almostforgotten and abandoned. If it is possible, by pressure, to cause the retention of these "two or three quarts of warm saline enema, until absorption shall take place, (and we are now for the first time told that the "absorbing veins of the colon are ready to take up whatever is presented to them of suitable proporties,") why not sooner have recourse to this all powerful agent, and by applying the pressure before the stools have become frequent—indeed, the very first thing—throw injections of lead and laudanum to the dogs, and abandon all treatment save that of holding the anus; for, by means simply of this almighty pressure can we not prevent the matter exhaled into the intestines from having exit? and can we not thus cause its re-absorption? It is nothing but the water and salts of the blood, and is certainly more suitable in its properties than any artificial substitute that human hands can prepare.

As a proof that absorption takes place readily and rapidly, Prof. A.'s patient has tasted enema "in from one to two minutes" after their administration. This is certainly rapid, and when we recollect that the matter tasted was first obliged to reach the liver, pass through its capillary system, go through the right side of the heart, through the pulmonic capillaries, back to the heart, and through its left cavities, the aorta, and vessels of the neck and head, before it could, by any possibility, on the absorbent principle, reach the nerves of taste, we are forced to admit that it was certainly quick work. Hereafter, we will never object to the expression "torrent of the circulation," for in comparison with it steamcars are antiquated sloths, and the electric telegraph a humbug.

"As far as I am acquainted with its properties, [the di-sulphate of quinine] I should suppose it to be contraindicated by the irritable state of the bowels." On this point, Prof. A. has considerably softened down since his advertisement of the 1st of August. In that he says: "I beg leave also to caution my friends against the use of sulphate of quinine. In the form of pill or powder, it is certain to act on the bowels if they are at all irritable, and cause a relapse. Even in the solution, in most cases I regard it as an unsafe remedy.

* Moseley, on Tropical Diseases, alludes to its employment by Septalius, whose work, in which anal tamponnement is recommended, was published in 1629. See also an article by Dr. T. S. Bell, published in the Transylvania Journal of Medicine, Vol. v., No. 4, 1832, in which the writer administered a severe, but well merited castigation to Dr. Clanny for pursuing the same practice. See also a well written and burlesque account of this same nonsensical idea, in the Gazette des Hôpitaux for April 24, 1849.

Until reading the above paragraph, we were not aware that quinine was remarkable for its purgative qualities. We knew that when given in inordinate doses, at least so say the authorities, it occasionally causes purgation, and we can readily believe that there are some few persons, who, owing to a peculiar idiosyncrasy, will be purged by it. But certainly this does not exist to so great an extent as to cause its rejection in all cases of irritable bowels, and most particularly not in a disease like epidemic cholera, of which prostration and depression of the vital power is so striking a characteristic. Of the many who have used it, we will only refer to Mr. Serres, who, in a paper* read before the Institute of France, recommends the following injection for the purpose of checking the bowels: starch, camphor, laudanum and quinine. We have referred to M. Serres alone, because his mode of using this article and the reason he assigns for its use, (its sedative effect upon the bowels) are directly and unequivocally opposed to the assertion of its irritating influence.

"The liver, when properly excited by calomel, after the stoppage of the serous hemorrhage from the intestines, has never refused to secrete bile, under my observation."

We ask for information. Does not the liver at all times, "when properly excited by calomel," secrete bile? Is not the secretion of bile, after the administration of a dose of calomel, the only evidence which we possess that the medicine has excited the liver? According to Prof. A.'s opinion that the blood is the seat of the disease, that "serous hemorrhage" is the great enemy to be combatted, should not the arrestation and supply of this loss of the watery particles, be of itself sufficient to set the liver to its proper work, and to restore equilibrium to the functions of all the organs? It does seem to us that only one answer can be given to each and all of the questions, though, as we said before, we only ask for information.

"I therefore claim originality not only for the first idea of the value of this potent agent [acetate of lead] but also for the best mode of using it."

On referring to Pereira's able and standard work, though we cannot learn when this salt was first used, we find that it was employed for medical purposes as early as the thirteenth century. But possibly the physicians of that and every succeeding age used it empirically, without the slightest knowledge of its value. As yet no work upon materia medica, so antiquated, has fallen under our eyes, as to contain no men-

---

* Gazette des Hôpitaux, April 10.

tion of this valuable medicine, and a tolerable description of its powers. So, in claiming "originality" "for the first idea of the value of this potent agent," the Professor must either be the accursed wanderer upon earth until the second advent, or else most unblushingly ignorant of the history of his profession. True, he alludes to its use by Drs. Graves and Bardsley, and he might have extended the list over sheets of foolscap, but in the next breath claims "originality" "in the first idea of the value" of a medicine so old that its origin is almost, if not quite, lost in the gloom of ages long past.

He also claims "originality" "for the best mode of administering it.' In Mosely on Tropical Diseases, page 404, we read as follows: "For an inveterate and harassing tenesmus, succeeding long continued dysenteries and diarrheas where bloody mucus, or sometimes blood, and sometimes purulent matter, is perpetually voided, with intolerable soreness about the anus, I have known glysters remove the disease very soon: R. Extracti saturni, drachma; Aquæ puræ tepidæ, uncias octo. M.

And when the soreness is sufficiently abated, he recommends the following glyster: "R. Extracti saturni, drachma; Decocti corticis quercus, uncias octo. M."

Possibly Prof. A. may never have seen Mosely's work, but his use of the acetate of lead is referred to by the late Prof. Eberle, in his work on Materia Medica and Therapeutics, which, being much more modern, it is presumable he has seen. So here we have Mosely pilfering from Dr. A. his original idea of injections of lead, but, not satisfied with that, he most unblushingly combines with them, sometimes, an article containing tannin, as though chemical incompatibles were to him but as moonshine.* In addition to the recommendations of authors, how many practitioners of medicine are there, throughout the country, who, for years, have been in the habit of using it? Even here, around the Doctor, under his very eyes as it were, a large number of physicians were using it, and that extensively, in the treatment of cholera, long before he was known to have treated his first case, and this is susceptible of proof by the druggists of Lexington.

We will not express our opinion of the qualifications of a Professor of the Theory and Practice of Medicine, who, through the pages of a most respectable, and, we hope, widely circulated medical journal, gives utterance to such views, and so boldly lays claim to such discoveries.—

* See also Trousseau & Pidoux, vol. i., pp. 154—6.

Is he really as ignorant as he appears, or does he, knowing better himself, presume upon the ignorance and credulity of his readers? Does he, like some of his compeers, from the other side of the Alleghanies, believe that we of the West are still steeped in a literary darkness more overpowering, more impenetrable, than that physical darkness which, by an avenging God, was spread over Egypt?

Who was the first to use injections of lead and laudanum, we do not pretend to know, but so far as concerns Prof. A.'s claim, it is without foundation even in his own imagination. He might as well lay claim to originality in the application of the bandage immediately after parturition, or gravely assert that the speculum may be used in the virgin vagina without injury to the hymen. R. P. H.

